# Identification of Peripheral Blood mIR-128-3p Molecular Signature in Children with Autism Spectrum Disorder

**DOI:** 10.2478/bjmg-2026-0009

**Published:** 2026-10-14

**Authors:** TN Popov, SD Minchev, TI Vachev

**Affiliations:** Medical Center Spectar – Plovdiv; 107 Macedonia Street; Plovdiv, Bulgaria.; Department of Psychology; University of Plovdiv “Paisii Hilendarski”, 24 Tzar Asen Street; Plovdiv, Bulgaria.; Department of Human Anatomy and Physiology; University of Plovdiv “Paisii Hilendarski”, 24 Tzar Asen Street; Plovdiv, Bulgaria.; Department of Molecular Biology; University of Plovdiv “Paisii Hilendarski”, 24 Tzar Asen St.; Plovdiv, Bulgaria.

**Keywords:** miRNAs, autism spectrum disorder (ASD), qRT-PCR

## Abstract

MicroRNAs (miRNAs) are key post-transcriptional regulators of gene expression. Given the dynamic nature of the peripheral blood transcriptome, profiling circulating miRNAs in autism spectrum disorder (ASD) represents a promising strategy for identifying novel disease-associated biomarkers. Large-scale miRNA expression profiling was performed using the μParaflo™ miRNA microarray assay on pooled RNA samples from 23 individuals with ASD and 21 healthy controls. Differential expression analysis (p < 0.05) identified several dysregulated miRNAs in peripheral blood, including miR-128-3p. Microarray findings for miR-128-3p were validated by quantitative reverse transcription PCR (qRT-PCR), which confirmed its significant downregulation in the ASD group. Target gene analysis using miRWalk indicated that miR-128-3p exerts multilevel regulatory effects across diverse biological processes. Notably, predicted and validated targets included genes involved in neuronal development (e.g., *PTEN*) and epigenetic regulation (e.g., *DNMT1, RNASEN, DICER,* and *EIF2C2*). Receiver operating characteristic (ROC) curve analysis demonstrated significant diagnostic potential of miR-128-3p for ASD. Collectively, these findings support an integrative model of ASD as a heterogeneous neurodevelopmental disorder in which specific miRNAs, such as miR-128-3p, contribute to disease pathogenesis through modulation of gene expression networks.

## INTRODUCTION

Autism spectrum disorders (ASD) comprise a heterogeneous group of neurodevelopmental conditions affecting approximately 1% of the global population [1]. ASD is characterized by impairments in social communication and interaction, along with restricted and repetitive patterns of behavior, typically manifesting before the age of three years. It is well established that ASD has a strong genetic component. However, in nearly 70% of cases, the primary genetic, epigenetic, or other underlying causes remain unidentified [2]. The difficulty in determining specific genetic determinants in most ASD cases has been attributed to several factors, including complex interactions among multiple genes, the heterogeneous nature of ASD in which diverse etiological pathways converge into a common behavioral phenotype, and the contribution of epigenetic mechanisms independent of defined genetic mutations [3]. Although many of these factors have been described in affected individuals, their precise roles in ASD susceptibility and progression remain incompletely understood. Previous gene expression studies in ASD have frequently focused on identifying individual genes or small gene sets associated with the disorder. In families without a history of ASD or related phenotypes, the “sporadic autism” hypothesis has gained increasing support. This hypothesis suggests that de novo mutations may account for a substantial proportion of ASD risk. Alternatively, ASD pathogenesis may arise from alterations in gene regulatory mechanisms, including dysfunction or dysregulation of regulatory molecules such as microRNAs (miRNAs). MicroRNAs (miRNAs) are small (~24 nt) non-coding RNAs that modulate gene expression by recognizing target mRNAs through complementary base pairing. Acting as post-transcriptional negative regulators, miRNAs play essential roles in fundamental cellular processes and in numerous pathological conditions. The human genome encodes more than 2,500 miRNAs (miRBase v21), which participate in diverse regulatory pathways and form complex networks predicted to regulate over 50% of protein-coding genes (PCGs). Many miRNAs exhibit spatiotemporal, tissue-specific, and developmentally regulated expression patterns [4], suggesting specialized roles in fundamental biological processes such as development, proliferation, and differentiation. Accumulating evidence links dysregulated miRNA expression to a wide range of human diseases [5]. Because a single miRNA can regulate multiple mRNA targets—and a single mRNA can be targeted by multiple miRNAs—protein expression is fine-tuned through highly complex and coordinated regulatory networks. The emerging involvement of miRNAs in diverse pathological conditions has stimulated extensive research aimed at elucidating their roles in disease mechanisms, exploring their potential as diagnostic and prognostic biomarkers, and developing miRNA-based therapeutic strategies. Peripheral blood represents an attractive source for such investigations, as it may reflect both local and systemic gene expression alterations. Several central nervous system (CNS)-enriched miRNAs have been identified, including miR-128, which is preferentially expressed in neurons [6]. Downregulation of miR-128-3p has been reported in multiple malignancies, including glioblastoma, neuroblastoma, prostate cancer, lung cancer, and acute lymphocytic leukemia. In glioma, miR-128 has been shown to interact with Polycomb Repressor Complexes (PRCs) in glioma stem cells. Specifically, miR-128 directly targets SUZ12 mRNA, a core component of PRC2, and also regulates BMI1 expression, another PRC-associated protein involved in chromatin-mediated gene repression. Reduced BMI1 expression results in decreased glioma cell proliferation both in vitro and in vivo and diminishes the self-renewal capacity of glioma stem-like cells [7]. Disruption of nonsense-mediated decay (NMD) has been implicated in neurological disorders, underscoring the importance of this pathway in brain function. Notably, brain-enriched miR-128 represses NMD. During brain development and neuronal differentiation, miR-128 levels increase markedly, leading to partial repression of NMD and subsequent stabilization of transcripts that would otherwise be degraded [8]. By attenuating—but not abolishing—NMD activity, miR-128 modulates transcript abundance while preserving the pathway’s quality control function. Among its key targets are reelin (RELN), which regulates neuronal migration and synaptic organization, and doublecortin (DCX), expressed in neuronal progenitors and migrating immature neurons. Both genes play critical roles in neuronal migration through distinct mechanisms [9]. Collectively, these findings underscore the pivotal role of miR-128 in normal tissue development and function, particularly within the CNS, and suggest that its dysregulation may contribute to the pathogenesis of neurological disorders. A distinctive feature of miRNAs is their ability to cross the blood–brain barrier and their remarkable stability in body fluids, including blood and saliva [10]. Consequently, miRNA expression profiles in peripheral blood may mirror pathological processes occurring in the CNS and hold promise as minimally invasive biomarkers for CNS-related conditions. Although blood-based protein biomarkers for autism spectrum disorder (ASD) have been extensively explored for diagnostic and prognostic purposes [11], the regulatory complexity, stability, and developmental relevance of miRNAs position them as particularly compelling biomarker candidates. To our knowledge, the present study is the first to associate peripheral blood expression levels of miR-128-3p with ASD, highlighting its potential relevance to disease-related molecular pathways.

## MATERIALS AND METHODS

### Ethics Statement

The study protocol and written informed consent forms (ICFs) were approved by the Ethics Committee of the Medical University of Plovdiv. Written informed consent was obtained from the parents or legal guardians of all participating children. The Institutional Review Board of the Medical University–Plovdiv approved the use of peripheral blood samples for research purposes. All personal data were handled in strict accordance with confidentiality and data protection regulations.

### Participants

The study group consisted of 30 children diagnosed with autism spectrum disorder (ASD), recruited from the Medical University Hospital of Plovdiv and an autism center in Plovdiv. The diagnosis of ASD (DSM-IV-TR criteria, code 299.00) was established through a routine clinical interview, comprehensive psychiatric evaluation, and the Autism Diagnostic Interview–Revised (ADI-R). The control group comprised 25 typically developing children (TDC), matched to the ASD group by sex (p = 1.0) and age (p = 0.825). All participants in the ASD group were evaluated by qualified psychiatric specialists. None of the participants were receiving medication at the time of the study, and all had consumed a standard breakfast prior to blood sampling. Children with chronic medical conditions were excluded from the analysis. Demographic characteristics of the ASD and TDC cohorts are summarized in Table 1.

**Table 1.** Structure and some demographic features of the ASD and typically developing children (TDC) cohorts used in this study.

**Demographic**	**ASD (n=23)**	**TDC (n=21)**
Sex, M/F	18 male; 5 female	17 male; 4 female
Mean age/years	4.76	4.57
Age range/years	3 – 6	3 – 6

### Blood Collection and RNA Extraction

Peripheral blood samples (2.5 mL) from all subjects were collected in PAXgene Blood RNA Tubes (PreAnalytiX GmbH, Hombrechtikon, Switzerland), which contain a reagent that lyses blood cells and immediately stabilizes intracellular RNA in order to preserve the gene expression profile. Prior to extraction, the PAXgene tubes were thawed at room temperature for 8 hours. Total RNA was isolated from all samples using the PAXgene Blood miRNA Isolation Kit, which is designed for the purification of RNA enriched in small RNA species. The extracted RNA was treated with RNase-free DNase I (Promega) according to the manufacturer’s protocol. RNA concentration (absorbance at 260 nm) and purity (A260/A280 ratio) were measured using the Epoch Micro-Volume Spectrophotometer System (BioTek), and RNA integrity was assessed by agarose gel electrophoresis. The A260/A280 ratio ranged from 1.93 to 2.1 for all samples included in the subsequent analyses. Pooled samples were prepared by combining equal amounts of total RNA from each individual sample to obtain a final amount of 5 µg RNA. The pooled RNA samples were precipitated according to the requirements of the μParaflo™ miRNA microarray assay service. Specifically, one-tenth volume of 3 M sodium acetate (NaOAc), pH 5.2, and three volumes of 100% ethanol were added to each sample to reach a final volume of 400 µL. RNA aliquots were stored at −80 °C and shipped on dry ice.

### MicroRNA Expression Profiling (LC Sciences)

For large-scale miRNA expression profiling, a pooled RNA approach was employed. Equal amounts of total RNA from individual ASD (n = 30) and TDC (n = 25) samples were combined into two pooled samples. RNA integrity for each pool was assessed using the Agilent 2100 Bio-analyzer. The μParaflo™ miRNA microarray assay (LC Sciences, Houston, TX) was performed using a proprietary microfluidic platform based on the Sanger miRBase v18.0 database, enabling detection of 1,898 unique mature human miRNA sequences. The assay required 5 µg of total RNA per sample. Total RNA was size-fractionated using a YM-100 Microcon centrifugal filter (Millipore, Billerica, MA) to isolate small RNAs (~300 nt). These small RNAs were polyadenylated at the 3′ end using poly(A) polymerase, followed by ligation of an oligonucleotide tag to facilitate subsequent fluorescent labeling. Hybridization was carried out overnight on a μParaflo microfluidic chip. Each probe on the array consisted of a chemically modified nucleotide sequence complementary to one of the 1,898 miRNAs listed in miRBase (Sanger Institute, Cambridge, U.K.). Internal control probes were included to monitor chip quality, labeling efficiency, and hybridization conditions. Hybridization was performed in 100 µL of 6× SSPE buffer (0.90 M NaCl, 60 mM Na_2_HPO_4_, 6 mM EDTA, pH 6.8) containing 25% formamide at 34 °C. Following hybridization, a Cy5 dye conjugated to the oligonucleotide tag was circulated through the chip for staining. Fluorescence signals were acquired using a GenePix 4000B laser scanner (Molecular Devices, Sunnyvale, CA) and quantified with Array-Pro image analysis software (Media Cybernetics, Bethesda, MD).

### Microarray Data Analysis

Microarray data analysis included the determination of detectable signals, calculation of signal intensities, and calculation of differential expression ratios (log_2_-transformed). Data processing consisted of background subtraction, detectability determination, and p-value calculation.

### RT-qPCR Analysis of Differentially Expressed miR-128-3p

Quantitative real-time PCR (RT-qPCR) was used to measure the expression of miR-128-3p in the ASD and TDC groups. cDNA was synthesized from 1 µg of total RNA using the RevertAid First Strand cDNA Synthesis Kit (Thermo Fisher Scientific) and miRNA-specific stem-loop primers. Five µL of the newly synthesized cDNA was pre-amplified prior to the qRT-PCR step to enhance assay sensitivity. The pre-amplification was performed in a 25 µL reaction volume containing 5 µL miRNA-specific cDNA mix, 2.5 µL 10× Long-Range PCR buffer (+Mg), 1.25 µL 10 mM dNTP mix, 2 µL pre-amplification primer mix (5 mM each), 0.2 µL Long-Range PCR enzyme mix, and nuclease-free water. qRT-PCR was conducted using an ABI PRISM 7500 system (Applied Biosystems) with Maxima SYBR Green qPCR Master Mix (Thermo Fisher Scientific). All PCR reactions were performed in 96-well plates in triplicate. A negative control (no-template control, NTC) was included in each experiment to monitor potential contamination. Ct values were selected within the linear range of amplification. Relative miRNA expression levels were calculated using the comparative 2^–ΔΔCt method [12]. Amplification of a single specific miR-128-3p product was confirmed by dissociation (melting) curve analysis and agarose gel electrophoresis. The used qRT-PCR primers were as follows: miR-128-3p Stem-loop RT primer: 5′ CTCAACTGGTGTCGTGGAGTCGGCAATTCAGTTGAGAAAAGAGA-3′; miR-128-3p PCR primers: Forward 5′-ACACTCCAGCTGGGTCACAGTGAACCGGTC-3′, Reverse 5′-TGGTGTC GTGGAGTCG-3′; U6 Stem-loop RT primer: 5′-GTCGTATCCAGTGCAGGGTCCGAGGTA TTCGCACTGGATACGACAAAATATGGAAC-3′; U6 PCR primers: sense: 5′-CTCGCTTCGGCAGCACA-3′, anti-sense: 5′- TGCAGGGTCCGAGGT -3′.

### MicroRNA Target Genes Analysis

Validated miRNA target genes were identified using the publicly available miRWalk database 2.0 database: http://zmf.umm.uni-heidelberg.de/apps/zmf/mirwalk2/ . The miRWalk database supports a direct search functionality only for pathways involving putative but not validated target genes. Thus, we developed a custom tool that takes the initial lists of target genes as an input and then automatically explores the KEGG database, for pathways enlisting the previously obtained validated miRNA targets. Subsequently, we plotted the total number of validated target genes with respect to the pathways in which they participate to construct the cmplete list of biological processes associated with the validated target genes of miR-128-3p (Table 2).

**Table 2.** Complete list of biological processes associated with the validated target genes of miR-128-3p.

**Biological Process**	**Genes**
GO:0007610~**behavior**	NLGN3, HRAS, MAOA, TSC2, SCN1A, OXTR, AFF2, KRAS, PAFAH1B1, COMT, GRIK2, KCNMA1, GABRA5, BDNF, AVPR1A, MET, DBH, CHRNA4, PTEN, BCL2, NF1, IL1RAPL1, GRPR, MAP2K1, CACNA1C, RELN, NLGN4X, TBX1, FOXP2, MKKS, AGTR2, CHRNB2, DRD3, DRD4, FEZF2, CHD7, ACSL4, MAPK1
GO:0019226~**transmission of nerve impulse**	GABRB3, NLGN3, MAOA, DMPK, SCN1A, OXTR, PAFAH1B1, COMT, GRIK2, KCND2, KCNMA1, GABRA5, NTF3, DBH, DMD, NRXN1, CHRNA4, NF1, GAD1, CACNA1E, CACNA1C, PAH, ALDH5A1, TSC1, SLC6A4, CHRNB2, CNTNAP2, DRD3, DRD4, GABRG3, MAPK1
GO:0007611~**learning or memory**	HRAS, PTEN, NLGN3, NF1, IL1RAPL1, GRPR, CACNA1C, OXTR, AFF2, PAFAH1B1, KRAS, COMT, FOXP2, BDNF, GABRA5, CHRNB2, DRD3, DBH, ACSL4
GO:0007268~**synaptic transmission**	GABRB3, NLGN3, MAOA, DMPK, OXTR, PAFAH1B1, COMT, GRIK2, KCND2, KCNMA1, GABRA5, NTF3, DBH, DMD, NRXN1, CHRNA4, GAD1, CACNA1E, CACNA1C, PAH, ALDH5A1, SLC6A4, CHRNB2, DRD3, DRD4, GABRG3, MAPK1
GO:0030030~**cell projection organization**	PTPN11, TSC2, PAFAH1B1, L1CAM, ARX, HOXA1, CYFIP1, OPHN1, CEP290, FGD1, FEZF1, BDNF, NTF3, FOXG1, TBR1, RPGRIP1L, DCX, DMD, NRXN1, PTEN, BCL2, MAP2K1, CACNA1F, RELN, TSC1, MKKS, SEMA5A, CHRNB2, FEZF2
GO:0048666~**neuron development**	RPE65, PTPN11, PAFAH1B1, L1CAM, HOXA1, ARX, CYFIP1, OPHN1, CEP290, FEZF1, BDNF, NTF3, FOXG1, TBR1, DCX, DMD, NRXN1, PTEN, BCL2, MAP2K1, CACNA1F, RELN, SEMA5A, EN2, CHRNB2, CNTNAP2, FEZF2
GO:0032990~**cell part morphogenesis**	NRXN1, DMD, BCL2, PTPN11, MAP2K1, PAFAH1B1, CACNA1F, RELN, L1CAM, ARX, HOXA1, MKKS, CYFIP1, SEMA5A, OPHN1, FEZF1, BDNF, CHRNB2, NTF3, FOXG1, RPGRIP1L, TBR1, FEZF2, DCX
GO:0050877~**neurological system process**	GABRB3, HRAS, RPE65, NLGN3, MAOA, DMPK, AFF2, OXTR, SCN1A, KRAS, PAFAH1B1, COMT, GRIK2, KCND2, KCNMA1, GABRA5, BDNF, NTF3, DBH, NRXN1, DMD, CHRNA4, PTEN, NF1, GAD1, IL1RAPL1, CACNA1E, GRPR, CACNA1C, CACNA1F, ALDH7A1, PAH, ALDH5A1, TBX1, FOXP2, MKKS, TSC1, AGTR2, SLC6A4, CHRNB2, CNTNAP2, DRD3, NDP, DRD4, GABRG3, CHD7, ACSL4, MAPK1
GO:0048812~**neuron projection morphogenesis**	NRXN1, DMD, PTPN11, BCL2, MAP2K1, PAFAH1B1, CACNA1F, RELN, L1CAM, ARX, HOXA1, CYFIP1, SEMA5A, OPHN1, FEZF1, BDNF, CHRNB2, NTF3, FOXG1, TBR1, FEZF2, DCX
GO:0030900~**forebrain development**	NF1, OXTR, PAFAH1B1, RELN, ARX, FOXP2, MKKS, TSC1, ATRX, OTX1, SLC6A4, FEZF1, CHRNB2, PITX1, YWHAE, FOXG1, RPGRIP1L, AVPR1A, FEZF2
GO:0031175~**neuron projection development**	NRXN1, DMD, PTEN, BCL2, PTPN11, MAP2K1, PAFAH1B1, CACNA1F, RELN, L1CAM, ARX, HOXA1, CYFIP1, SEMA5A, OPHN1, FEZF1, BDNF, CHRNB2, NTF3, FOXG1, TBR1, DCX, FEZF2
GO:0035176~**social behavior**	NLGN3, TBX1, MKKS, GRPR, OXTR, CHRNB2, DRD3, DRD4, AVPR1A, NLGN4X
GO:0007409~**axonogenesis**	NRXN1, BCL2, PTPN11, PAFAH1B1, CACNA1F, RELN, L1CAM, ARX, HOXA1, CYFIP1, SEMA5A, OPHN1, FEZF1, BDNF, CHRNB2, NTF3, FOXG1, TBR1, FEZF2, DCX
GO:0030182~**neuron differentiation**	RPE65, PTPN11, PAFAH1B1, L1CAM, HOXA1, KCNMA1, ARX, CYFIP1, OPHN1, CEP290, FEZF1, BDNF, NTF3, FOXG1, TBR1, DCX, DMD, NRXN1, PTEN, BCL2, MAP2K1, CACNA1F, RELN, SEMA5A, EN2, CHRNB2, CNTNAP2, FEZF2
GO:0007267~**cell-cell signaling**	GABRB3, NLGN3, MAOA, DMPK, OXTR, PAFAH1B1, COMT, GRIK2, KCNMA1, KCND2, IGF2, BDNF, GABRA5, NTF3, DBH, DMD, CHRNA4, GAD1, NRXN1, CACNA1E, CACNA1C, PAH, ALDH5A1, SEMA5A, SLC6A4, CHRNB2, RAPGEF4, DRD3, NDP, DRD4, GABRG3, MAPK1
GO:0048667~**cell morphogenesis involved in neuron differentiation**	NRXN1, BCL2, PTPN11, PAFAH1B1, CACNA1F, RELN, L1CAM, ARX, HOXA1, CYFIP1, SEMA5A, OPHN1, FEZF1, BDNF, CHRNB2, TBR1, FOXG1, NTF3, FEZF2, DCX
GO:0000902~**cell morphogenesis**	NRXN1, BCL2, DMD, MAP2K1, PTPN11, PAFAH1B1, CACNA1F, RELN, L1CAM, ARX, HOXA1, MKKS, CYFIP1, SEMA5A, OPHN1, FEZF1, BDNF, CHRNB2, NTF3, FOXG1, RPGRIP1L, TBR1, FEZF2, DCX
GO:0032989~**cellular component morphogenesis**	PTPN11, PAFAH1B1, L1CAM, HOXA1, ARX, CYFIP1, OPHN1, FEZF1, BDNF, TTN, NTF3, FOXG1, TBR1, RPGRIP1L, DCX, NRXN1, DMD, BCL2, MAP2K1, CACNA1F, RELN, MKKS, SEMA5A, CHRNB2, FEZF2
GO:0000904~**cell morphogenesis involved in differentiation**	NRXN1, BCL2, PTPN11, PAFAH1B1, CACNA1F, RELN, L1CAM, ARX , HOXA1, CYFIP1, SEMA5A, OPHN1, FEZF1, BDNF, CHRNB2, NTF3, FOXG1, TBR1, FEZF2, DCX
GO:0051705~**behavioral interaction between organisms**	NLGN3, TBX1, MKKS, GRPR, OXTR, CHRNB2, DRD3, DRD4, AVPR1A, NLGN4X
GO:0007612~**learning**	HRAS, NLGN3, FOXP2, NF1, CACNA1C, GABRA5, CHRNB2, KRAS, DRD3, COMT, DBH
GO:0044057~**regulation of system process**	HRAS, NLGN3, PTPN11, DMPK , MAP2K1, CACNA1C, OXTR, KRAS, GRIK2, IGF2, KCNMA1, AGTR2, BDNF, CHRNB2 , NTF4, DRD3, NTF3, DRD4, AVPR1A, DBH
GO:0021537~**telencephalon development**	ARX, FOXP2, NF1, TSC1, MKKS, OXTR, PAFAH1B1, YWHAE, AVPR1A, RPGRIP1L, RELN
GO:0007411~**axon guidance**	NRXN1, L1CAM, RELN, HOXA1, ARX, SEMA5A, OPHN1, BDNF, FEZF1, NTF3, FOXG1, TBR1, FEZF2
GO:0008542~**visual learning**	HRAS, NLGN3, NF1, CACNA1C, CHRNB2, KRAS, DRD3, DBH
GO:0048878~**chemical homeostasis**	CHRNA4, NLGN3, BCL2, NF1, PTPN11, DMPK, CACNA1E, PRKCB, CACNA1C, OXTR, SCN1A, CACNA1F, GRIK2, IGF2, KCND2, KCNMA1, TSC1, SLC9A6, CHRNB2, NTF3, DRD3, DRD4, AVPR1A, DBH
GO:0006928~**cell motion**	NRXN1, DNAH5, PTEN, MAP2K1, PAFAH1B1, RELN, L1CAM, ARX, HOXA1, TBX1, SEMA5A , OPHN1, FEZF1, BDNF, NTF3Y, YWHAE, FOXG1, TBR1, SATB2, MET, DBH, FEZF2, DCX
GO:0042391~**regulation of membrane potential**	CHRNA4, NLGN3, BCL2, NF1, DMPK, SCN1A, GRIK2, KCND2, KCNMA1, TSC1, CHRNB2, NTF3, DRD4
GO:0007632~**visual behavior**	HRAS, NLGN3, NF1, CACNA1C, CHRNB2, KRAS, DRD3, DBH
GO:0030534~**adult behavior**	CHRNA4, KCNMA1, CACNA1C, SCN1A, PAFAH1B1, CHRNB2, DRD3, DRD4, MET, DBH, CHD7
GO:0050801~**ion homeostasis**	CHRNA4, NLGN3, BCL2, NF1, DMPK, PRKCB, CACNA1C, OXTR, SCN1A, CACNA1F, GRIK2, KCND2, KCNMA1, TSC1, SLC9A6, CHRNB2, NTF3 , DRD3, DRD4, AVPR1A
GO:0033555~**multicellular organismal response to stress**	GRIK2, BCL2, BDNF, GABRA5, DRD4, COMT, RELN, DBH
GO:0006873~**cellular ion homeostasis**	CHRNA4, NLGN3, BCL2, NF1, DMPK, PRKCB, CACNA1C, OXTR, SCN1A, CACNA1F, GRIK2, KCND2, KCNMA1, TSC1, CHRNB2, DRD3, NTF3, DRD4, AVPR1A
GO:0055082~**cellular chemical homeostasis**	CHRNA4, NLGN3, BCL2, NF1, DMPK, PRKCB, CACNA1C, OXTR, SCN1A, CACNA1F, GRIK2, KCND2, KCNMA1, TSC1, DRD3, CHRNB2, NTF3, DRD4, AVPR1A

### Statistical Analysis

All statistical analyses were performed using SPSS software, version 18.0 (SPSS Inc., Chicago, IL, USA). The distribution of qRT-PCR data was assessed for normality using the Kolmogorov–Smirnov test. As the data did not meet the assumptions of normal distribution, statistical comparisons between groups were performed using the Mann–Whitney U test. A p-value < 0.05 was considered statistically significant.

### Receiver Operating Characteristic (ROC) Analysis of miR-128-3p in ASD

To evaluate the diagnostic potential of miR-128-3p as a biomarker for ASD, receiver operating characteristic (ROC) curve analysis was performed using MedCalc software, version 9.6.4 (https://www.medcalc.org/). The area under the ROC curve (AUC) was calculated to assess diagnostic accuracy.

### Experimental pipeline

A process timeline chart providing an overview of the utilized experimental procedures for miRNA expression profiling is shown in **Figure 1.**

**Figure 1.**
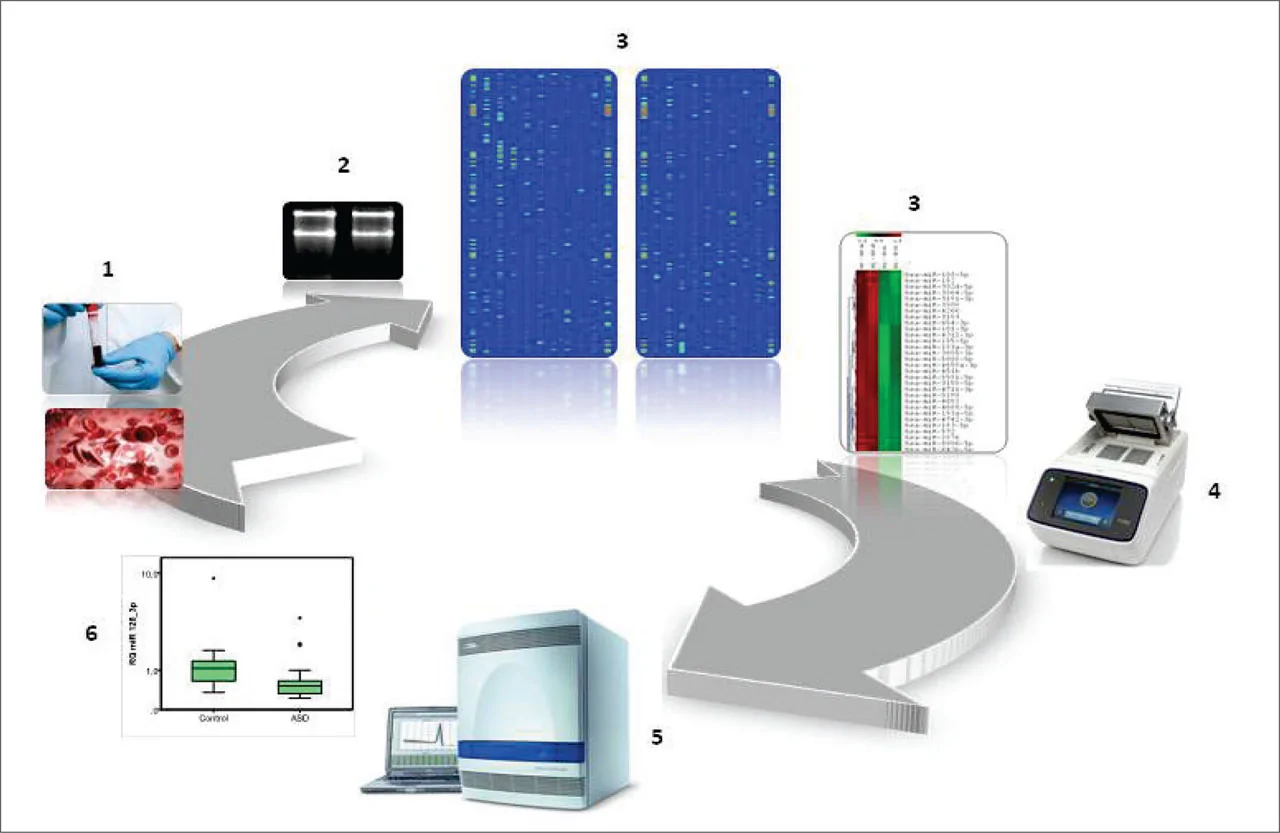
**Process flowchart.** 1) Patient enrolment and blood collection; 2) Total RNA extraction for downstream analyses; 3) MicroRNA Expression Profiling (LC Science); 4) Stem-loop Reverse Transcription of miRNAs; 5) miRNA specific Real-Time qPCR; 6) Data analysis using the SPSS software.

## RESULTS

### Micro RNA Microarray assay (LC Sciences)

Using μParaflo^TM^ miRNA microarray assay based on the Sanger miRBase v18.0 database, we found a set of differentially expressed miRNAs (*p* < 0,05) between the ASD and TDC groups, including: miR-766-3p, log_2_ -1.10; miR-128-3p, log_2_ - 0.97; miR-29a-3p, log_2_ - 0.67; miR-361-5p, log_2_ - 0.70; miR-148a-3p, log_2_ - 0.96; and miR-663, log_2_ - 0.10. There was a higher amount of miRNAs with decreased expression than overexpression in the ASD sample, which implies dysfunction of specific miRNAs, resulting in accumulation of the targeted proteins and their functions. Out of the six microRNA molecules analyzed, only miR-128-3p was confirmed to be differentially expressed, while the expression of the remaining five miRNAs remains unverified.

### Confirmation of the differentially expressed miR-128-3p in case-control pairs by qRT-PCR analysis

To validate the results of the miRNA microarray assay, we examined the expression of miR-128-3p in all individual cases by stem-loop qRT-PCR, using RNA samples from the subjects in the ASD and TDC control groups. This miRNA was selected due to its involvement in CNS-related processes as mentioned above. Expression levels of miR-128-3p were assessed with the Mann–Whitney test and normalized to endogenous U6 snRNA. The results showed a significant down-regulation (relative expression of 0.67) of miR-128-3p in the ASD, compared to the TDC group, which matches the data from the microarray experiment (**Figure 2**). The observed downregulation in ASD patients was statistically significant (*p* = 0.028) (**Figure 2).**

**Figure 2.**
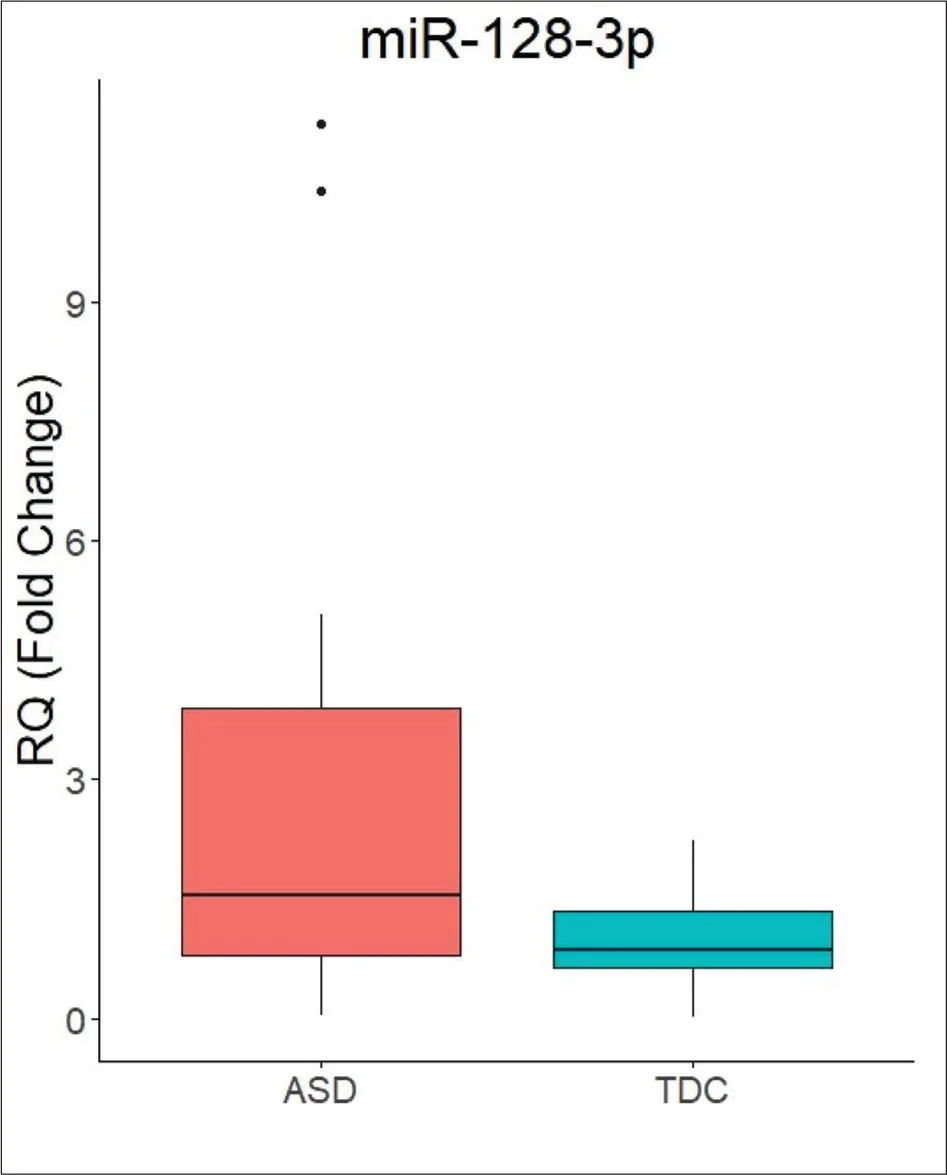
A box plot diagram showing the miR-128-3p expression profile determined by qRT-PCR in individual ASD and control (TDC) samples. The top of the box indicates the 75th percentile while the bottom corresponds to the 25th percentile. The line in the middle represents the median. Circles and the asterisks represent the outliers.

### Evaluation of the Diagnostic Potential of miR-128-3p for ASD

To evaluate the strength of miR-128-3p as potential blood biomarkers for ASD, a Receiver Operating Characteristic (ROC) curve analysis was performed with the data from the qRT-PCR experiment. Subsequently, the area under the ROC curve (AUC), as well as the diagnostic sensitivity and specificity of miR-128-3p. were calculated and presented in **Figure 3**. The AUC was calculated to be 0.694. When evaluated as a standalone predictor of ASD, miR-128-3p exhibited 0.61 sensitivity and 0.60 specificity, with a standard error of 0.081 a 95% confidence interval ranging from 0.533 to 0.853, and *p* = 0.017. This evidence highlights the diagnostic relevance of miR-128-3p for ASD and positions it as a viable blood-based biomarker for the condition.

**Figure 3.**
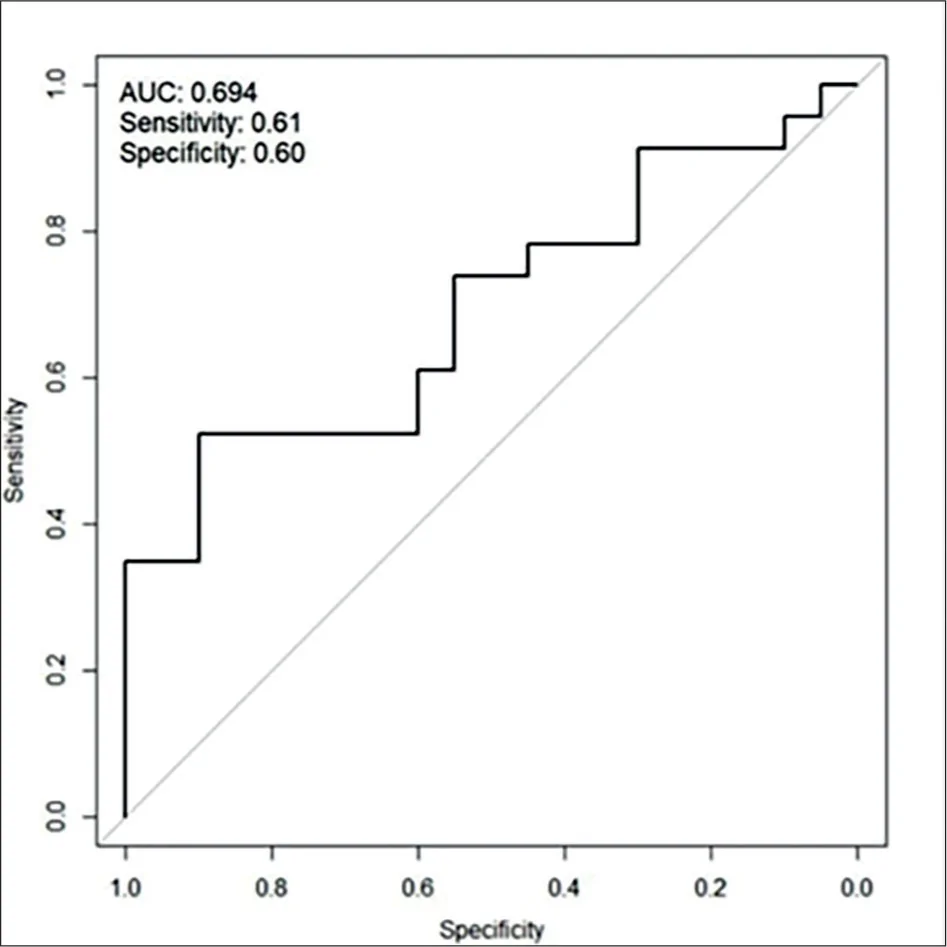
Performance of the miR-128-3p prediction model. Receiver operating characteristic (ROC) curve analysis of miR-128-3p was conducted to assess its predictive accuracy for ASD. The AUC was calculated to be 0.694. The diagonal lines on the plots represent the random classification accuracy (AUC 0.5).

### Target Gene Analysis of miR-128-3p and Its Possible Role in ASD

A detailed list of validated protein-coding gene (PCG) targets of the dysregulated miR-128-3p was obtained from the miRWalk 2.0 database: http://zmf.umm.uni-heidelberg.de/apps/zmf/mirwalk2/. The identified targets include: MLL3 (lysine-specific methyltransferase 2C), a member of the ASC-2/NCOA6 complex (ASCOM), which possesses histone methyltransferase activity and participates in transcriptional coactivation. DNMT1 (DNA (cytosine-5)-methyltransferase 1), an enzyme involved in the establishment and maintenance of tissue-specific DNA methylation patterns. ARPP21 (cAMP-regulated phosphoprotein, 21 kDa), a protein enriched in the caudate nucleus and cerebellar cortex; its murine homolog is implicated in modulating dopaminergic signaling within the basal ganglia. ADORA2B (adenosine A2B receptor), a G protein–coupled receptor that interacts with netrin-1, a molecule involved in axonal elongation. RNASEN (Drosha, RNase III), the core nuclease responsible for initiating microRNA (miRNA) processing in the nucleus. SNAP25 (synaptosomal-associated protein, 25 kDa), a presynaptic plasma membrane protein essential for the regulation of neurotransmitter release. DNAI1 (dynein axonemal intermediate chain 1), mutations of which lead to abnormal ciliary ultrastructure and are associated with primary ciliary dyskinesia and Kartagener syndrome. DNAI1 may also contribute to dendritic development and rapid cytoskeletal reorganization within dendritic spines during synaptic plasticity. Additionally, defects in DNAI1 have been linked to X-linked intellectual disability (MRX30/MRX47). EIF2C2 (Argonaute RISC catalytic component 2), which interacts with DICER1 and plays a central role in RNA interference–mediated gene silencing. NTRK3 (neurotrophic tyrosine kinase receptor type 3), whose signaling promotes cell differentiation and the development of proprioceptive neurons. Another notable target of miR-128-3p is PTEN (phosphatase and tensin homolog), a tumor suppressor that negatively regulates intracellular phosphatidylinositol-3,4,5-trisphosphate levels and inhibits the AKT/PKB signaling pathway. Importantly, comparison of our validated miRNA–PCG target dataset (see Materials and Methods) with de novo risk-contributing mutations identified in exome sequencing studies of ASD patients [14] revealed an overlap with a single gene, PTEN. Potentially damaging de novo mutations in PTEN were detected in 0.35% of ASD patients screened by O’Roak et al. [14]. Collectively, these findings support a cumulative model for the development and progression of ASD, involving the interplay between genetic background including de novo mutations and dysregulation of miRNA–PCG expression networks.

## DISCUSSION

The identification of key molecules that epigenetically transduce diverse stress signals into stable pathological patterns would facilitate the development of preventive and disease-modifying therapeutics for neurological disorders. Previous studies have reported differentially expressed genes in peripheral blood samples of patients with ASD [15]. Applying such findings to prospective cohorts would enable further validation of their diagnostic and prognostic value. Global microRNA expression profiling represents one of the most effective strategies for identifying aberrantly expressed miRNAs in disease states. Peripheral blood is considered an appropriate surrogate tissue, as it may reflect responses to both immediate and distant environmental changes through alterations in miRNA expression levels. For molecular diagnostic purposes, the development of blood-based biomarkers for ASD is particularly desirable, given that whole blood collection is minimally invasive and suitable for large patient cohorts, including individuals in the early stages of disease manifestation. Furthermore, several studies indicate that blood gene expression profiles are enriched for brain-expressed genes, suggesting that peripheral blood may serve as a source of non-invasive biomarkers for brain-related disorders [16, 17]. In the present study, two independent technical platforms were employed to assess the differential expression of the neuronally enriched miR-128-3p in peripheral blood samples from ASD probands. A key finding was the significant downregulation of miR-128-3p in ASD patients, confirmed through the analysis of all individual samples across the two study groups. MiR-128-3p has also been implicated in other pathological conditions. For instance, numerous studies have demonstrated that several miRNAs, including miR-128, are downregulated in various tumors compared to normal tissues [18]. The ability of a single microRNA such as miR-128-3p to simultaneously regulate a broad spectrum of mRNA targets with potential pro-oncogenic properties highlights a widespread miRNA-mediated modulatory mechanism affecting biological pathways relevant to multiple disorders. In the context of ASD, a miRNA-centered approach aimed at identifying specific miRNAs capable of fine-tuning global gene expression may provide insight into the molecular basis of the disorder and contribute to the discovery of promising biomarker candidates. Bioinformatic tools are currently invaluable for identifying miRNA binding sites within protein-coding genes of potential clinical relevance. However, future diagnostic strategies may benefit from investigating combined miRNA–protein-coding gene (miRNA:PCG) pairs rather than individual molecules. Analysis of validated target genes revealed that many have previously been associated with ASD (according to AutismKB). The complete list of biological processes in which these validated target genes participate is presented in Table 2. Several validated miR-128-3p target genes encode proteins that directly interact with one another, suggesting coordinated involvement in common biological processes such as synaptic transmission, neuronal development, axonogenesis, neuron projection morphogenesis, axon guidance, and nerve impulse transmission. These findings support a multilevel mode of action of miR-128-3p, influencing different stages of interconnected neurobiological pathways. Among the miR-128-3p target genes implicated in ASD-relevant biological processes are NLGN3 (associated with behavior, nerve impulse transmission, synaptic transmission, and social behavior), RELN (involved in neuron projection development, axon guidance, and cell migration), and DCX (associated with axonogenesis and cell morphogenesis during differentiation) (see Table 2 for detailed annotation). Germline mutations in the nonsense-mediated mRNA decay (NMD) gene UPF3B have been shown to cause intellectual disability [19], and subsequent studies have linked UPF3B mutations to autism, attention-deficit/hyperactivity disorder (ADHD), and schizophrenia [20]. UPF3B is thought to regulate mRNA expression and degradation at synapses. Given that miR-128 has been reported to repress the NMD pathway, and that NMD dysregulation may disrupt biological processes associated with ASD, it is plausible that miR-128-3p dysregulation and related pathway alterations contribute to ASD etiology. The biological mechanisms underlying miR-128-3p dysregulation, the downstream pathways affected, and the full diagnostic potential of this miRNA require further investigation. Future molecular studies examining miRNA and target gene expression in larger cohorts will provide a more comprehensive understanding of ASD pathophysiology. In conclusion, this study identifies a specific peripheral blood miR-128-3p expression signature with promising discriminatory power between children with ASD and typically developing controls. These findings support the potential of miR-128-3p as a prognostic biomarker for ASD and represent a meaningful step toward elucidating its functional role in ASD etiology. Larger-scale studies are warranted to determine whether ASD can be reliably predicted based on miRNA expression signatures.

## References

[1] Abrahams B.S., Geschwind D.H. (2008). Advances in autism genetics: on the threshold of a new neurobiology. Nature Reviews Genetics.

[2] Schaaf C.P., Zoghbi H.Y. (2011). Solving the autism puzzle a few pieces at a time. Neuron.

[3] Stephan D.A. (2008). Unraveling autism. American Journal of Human Genetics.

[4] He L., Hannon G.J. (2004). MicroRNAs: small RNAs with a big role in gene regulation. Nature Reviews Genetics.

[5] Zimmerman A.L., Wu S. (2011). MicroRNAs, cancer and cancer stem cells. Cancer Letters.

[6] Smirnova L., Gräfe A., Seiler A., Schumacher S., Nitsch R. (2005). Regulation of miRNA expression during neural cell specification. European Journal of Neuroscience.

[7] Godlewski J., Nowicki M.O., Bronisz A., Williams S., Otsuki A. (2008). Targeting of the Bmi-1 oncogene/stem cell renewal factor by microRNA-128 inhibits glioma proliferation and self-renewal. Cancer Research.

[8] Sempere L.F., Freemantle S., Pitha-Rowe I., Moss E., Dmitrovsky E. (2004). Expression profiling of mammalian microRNAs uncovers a subset of brainexpressed microRNAs with possible roles in murine and human neuronal differentiation. Genome Biology.

[9] Evangelisti C., Florian M.C., Massimi I., Dominici C., Giannini G. (2009). MiR-128 up-regulation inhibits Reelin and DCX expression and reduces neuroblastoma cell motility and invasiveness. The FASEB Journal.

[10] Hicks S.D., Ignacio C., Gentile K., Middleton F.A. (2016). Salivary miRNA profiles identify children with autism spectrum disorder, correlate with adaptive behavior, and implicate ASD candidate genes involved in neurodevelopment. BMC Pediatrics.

[11] Momeni N., Bergquist J., Brudin L., Behnia F., Sivberg B. (2012). A novel blood-based biomarker for detection of autism spectrum disorders. Translational Psychiatry.

[12] Livak K.J., Schmittgen T.D. (2001). Analysis of relative gene expression data using real-time quantitative PCR and the 2(-Delta Delta C(T)) method. Methods.

[13] Xu L.M., Li J.R., Huang Y., Zhao M., Tang X. (2012). AutismKB: an evidence-based knowledge base of autism genetics. Nucleic Acids Research.

[14] O’Roak B.J., Vives L., Fu W., Egertson J.D., Stanaway I.B. (2012). Multiplex targeted sequencing identifies recurrently mutated genes in autism spectrum disorders. Science.

[15] Kong S.W., Collins C.D., Shimizu-Motohashi Y., Holm I.A., Campbell M.G. (2012). Characteristics and Predictive Value of Blood Transcriptome Signature in Males with Autism Spectrum Disorders. Plos One.

[16] Jasinska A.J., Service S., Choi O.W., DeYoung J., Grujic O. (2009). Identification of brain transcriptional variation reproduced in peripheral blood: an approach for mapping brain expression traits. Human Molecular Genetics.

[17] Rollins B., Martin M.V., Morgan L., Vawter M.P. (2010). Analysis of whole genome biomarker expression in blood and brain. American Journal of Medical Genetics Part B Neuropsychiatric Genetics.

[18] Lu J., Getz G., Miska E.A., Alvarez-Saavedra E., Lamb J. (2005). MicroRNA expression profiles classify human cancers. Nature.

[19] Tarpey P.S., Raymond F.L., Nguyen L.S., Rodriguez J., Hackett A. (2007). Mutations in UPF3B, a member of the nonsense-mediated mRNA decay complex, cause syndromic and nonsyndromic mental retardation. Nature Genetics.

[20] Addington A.M., Gauthier J., Piton A., Hamdann F.F., Raymond A. (2011). A novel frameshift mutation in UPF3B identified in brothers affected with childhood onset schizophrenia and autism spectrum disorders. Molecular Psychiatry.

